# Reparative and Maladaptive Inflammation in Tendon Healing

**DOI:** 10.3389/fbioe.2021.719047

**Published:** 2021-07-19

**Authors:** Varun Arvind, Alice H. Huang

**Affiliations:** ^1^Department of Orthopedics, Icahn School of Medicine at Mount Sinai, New York, NY, United States; ^2^Department of Orthopedic Surgery, Columbia University, New York, NY, United States

**Keywords:** reparative inflammation, wound healing, tendon, tendinopathy, regeneration

## Abstract

Tendon injuries are common and debilitating, with non-regenerative healing often resulting in chronic disease. While there has been considerable progress in identifying the cellular and molecular regulators of tendon healing, the role of inflammation in tendon healing is less well understood. While inflammation underlies chronic tendinopathy, it also aids debris clearance and signals tissue repair. Here, we highlight recent findings in this area, focusing on the cells and cytokines involved in reparative inflammation. We also discuss findings from other model systems when research in tendon is minimal, and explore recent studies in the treatment of human tendinopathy to glean further insights into the immunobiology of tendon healing.

## Introduction

Tendons are connective tissues that facilitate movement by transmitting muscle forces to the skeleton. Tendon injuries can be caused by acute (i.e., laceration) or chronic (i.e., mechanical overuse) insults. While the term “tendinopathy” broadly defines any problem related to tendons, more specific terms such as tendinitis and tendinosis refer to tendon damage associated with inflammatory and non-inflammatory tendon degeneration, respectively (Millar et al., 2021). In recent years, an increase in recreational activities among middle-aged individuals has resulted in a large increase in the incidence of tendon injuries. Following injury, maladaptive tendon healing with scar tissue formation frequently leads to chronic pain and disability, with restoration of function occurring in only 60% of patients (Wu et al., 2017). An estimated 110 million Americans report musculoskeletal disability, with tendinopathy as the fourth leading cause of missed work among non-fatal diseases (American Academy of Orthopaedic Surgeons, 2016). Despite the high incidence of injury, repair strategies have seen little advancement due to the limited understanding of basic tendon biology and healing (Snedeker and Foolen, 2017).

In tendon healing, much of what is known has come from animal models of tendon injury. There remains significant controversy regarding the role of inflammation in tendinopathy and tendon healing (Paavola et al., 2002; Dean et al., 2016). Historically, the term tendinopathy is preferred to tendinitis, as several studies observed mucoid degeneration with a lack of inflammatory infiltrate in biopsy specimens from patients with chronic tendon disease, suggesting a pathology of tendinosis (Puddu et al., 1976; Aström and Rausing, 1995; Jozsa and Kannus, 1997; Khan et al., 1999). However, advancements in cellular profiling using genetic and molecular tools enabled research demonstrating the presence of mast cells, granulocytes, macrophages, T cells, and B cells in both acute and chronic human tendinopathic tissues (Rees et al., 2014; Dean et al., 2016). These data suggest a potential role for immune cells in tendon healing; however, little is known regarding how inflammation is orchestrated to either promote or impede tendon healing.

Here, we focus primarily on *in vivo* model systems in rodents and clinical studies performed in humans that test the role of inflammation in tendon healing. Where evidence from tendon is scarce, we also discuss findings from other tissues (e.g., muscle, lung, gut, brain, and skin) and highlight currents gaps in knowledge and opportunities for further research.

## Reparative Inflammation

**FIGURE 1:**
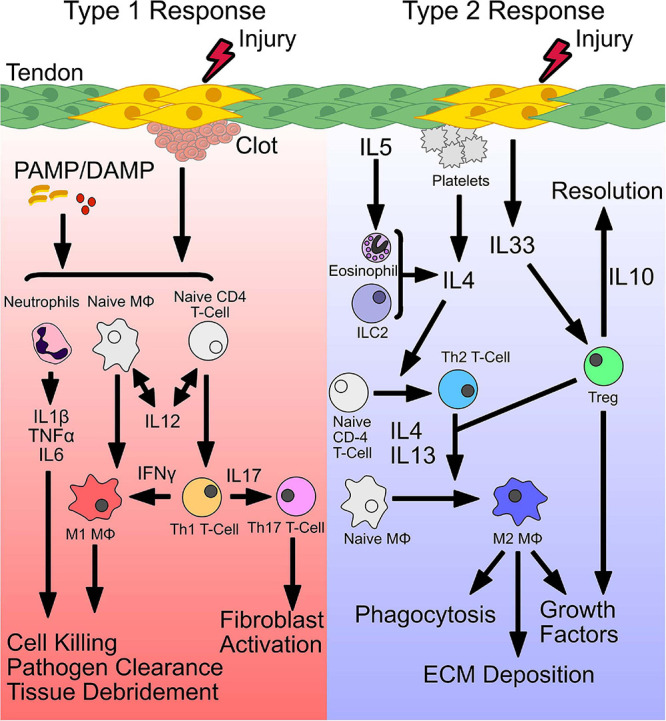
Initiation of a type 1 and type 2 immune response. Following injury, distinct signals establish a type 1 or type 2 immune response. Release of pathogen-associated or damage-associated molecular patterns (PAMP/DAMP), bind toll-like receptors (TLRs) and stimulate release of inflammatory cytokines. Secretion of IL-12, primes conversion of naive CD4 T cells and macrophages (Mϕ) to form Th1 and M1 phenotypes, that secrete IFNγ, to sustain a type 1 immune environment. Together, stimulated neutrophils and M1 macrophages perform pathogen killing and cellular debridement of dead or dying cells to facilitate deposition of new ECM and recruitment of cells. Polarized Th1 T cells also produce IL17, which stimulates Th17 T cells and fibroblast activation. Type 2 immunity is initiated by secretion of IL4 or release of the alarmin IL33 from damaged cells. In addition, secretion of IL4 from accessory cells including eosinophils, type 2 innate lymphoid cells (ILC2s), and Tregs can also induce a type 2 immune response. IL4 drives conversion of naive CD4 T cells and macrophages to a Th2 T cell or M2 macrophage phenotype, respectively. M2 macrophages act downstream to phagocytose tissue debris, deposit ECM, and secrete growth factors to stimulate stem and progenitor cells. In addition, stimulated Tregs produce IL10 which resolves type 1 inflammation.

While there is considerable interest in the cellular and molecular mechanisms that define proliferation, recruitment, and differentiation of tenocytes and other intrinsic cell types during healing (Dyment et al., 2013; Asai et al., 2014; Howell et al., 2017; Best and Loiselle, 2019; Harvey et al., 2019; Kaji et al., 2020), less is known regarding the preceding inflammatory stages. Inflammatory responses to wounding are highly orchestrated and evolutionarily conserved. An initial inflammatory response is required for successful tissue healing in a diverse set of species, including starfish, drosophila, zebrafish, axolotl, and mice (Metchnikov, 1893). Consequently, reparative inflammatory responses are hypothesized to have been co-opted from basic inflammatory responses to two distinct insults (Figure 1). In the first, an organism must respond rapidly to small pathogens including bacteria, fungi, and viruses, which can overcome the host through rapid proliferation (Figure 1). A rapid type I immune response is therefore deployed leading to the production of inflammatory cytokines TNFα, IFNγ, IL1β, and iNOS with recruitment of type I associated immune cells (Th1 T-cells, neutrophils, M1-macrophages). Together, this system elicits the cardinal signs of inflammation (rubor, tumor, calor, dolor) that promote pathogen destruction and clearance. Well described immunomodulatory stimuli including gram-negative endotoxin, lipopolysaccharide, and bacterial DNA directly result in production of IL1α, IL12, and IFNγ with subsequent recruitment and polarization to a type I inflammatory landscape (Skeen et al., 1996; Pisetsky, 1999; Spellberg and Edwards, 2001). In the second type of insult, migrating parasites that have breached mucosal barriers, produce chronic tissue damage (Figure 1). As these parasites complete the majority of their life cycle outside the host, a type II immune response that promotes tissue restoration is selected over a type I response (Gause et al., 2013). Cellular necroptosis and injury from parasitic infection stimulates the release of cytokines and alarmins that promote the production of type II polarizing (anti-inflammatory) cytokines including IL4, IL13, TGFβ, and IL10 (Romani et al., 1997; Gause et al., 2013; Mishra et al., 2019). Additionally, “frustrated phagocytosis” of large particle debris from foreign bodies signals the release of type II polarizing cytokines (Mishra et al., 2019). In the short term, this type II response promotes tissue repair, however, long term type II responses can promote encapsulation or fibrosis if severe.

Following injury, distinct signals establish a type 1 or type 2 immune response. Release of pathogen-associated or damage-associated molecular patterns (PAMP/DAMP), bind toll-like receptors (TLRs) and stimulate release of inflammatory cytokines. Secretion of IL-12, primes conversion of naive CD4 T cells and macrophages (MΦ) to form Th1 and M1 phenotypes, that secrete IFNγ, to sustain a type 1 immune environment. Together, stimulated neutrophils and M1 macrophages perform pathogen killing and cellular debridement of dead or dying cells to facilitate deposition of new ECM and recruitment of cells. Polarized Th1 T cells also produce IL17, which stimulates Th17 T cells and fibroblast activation. Type 2 immunity is initiated by secretion of IL4 or release of the alarmin IL33 from damaged cells. In addition, secretion of IL4 from accessory cells including eosinophils, type 2 innate lymphoid cells (ILC2s), and Tregs can also induce a type 2 immune response. IL4 drives conversion of naive CD4 T cells and macrophages to a Th2 T cell or M2 macrophage phenotype, respectively. M2 macrophages act downstream to phagocytose tissue debris, deposit ECM, and secrete growth factors to stimulate stem and progenitor cells. In addition, stimulated Tregs produce IL10 which resolves type 1 inflammation.

Therefore, emerging evidence supports the idea that a balance of type I and type II immune responses is critical for effective tissue repair and overactivation of either response can lead to pathogenesis (Eming et al., 2017). Consistent with this dogma, in tendon, systemic delivery of dexamethasone to suppress inflammation immediately following injury results in poor healing while delayed administration results in improved tendon healing in rats (Blomgran et al., 2017). These data suggest an early activation of type I inflammation followed by an anti-inflammatory type II response may improve tendon healing. In the following sections, we will discuss the cellular and molecular components of type I and type II immune responses and their respective roles in tendon healing. The following sections will discuss the relevant biology that can be gleaned from other tissues with respect to reparative inflammation (e.g., muscle, heart, gut, lung), and will also highlight studies conducted in tendon.

## Cells

**FIGURE 2:**
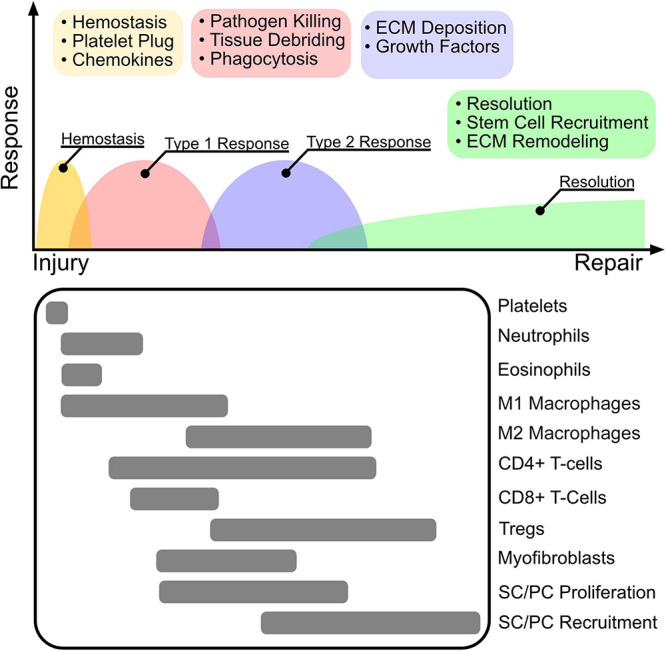
The wound healing cascade. The inflammatory response to wounding is highly orchestrated to follow four main programs after injury including hemostasis, type 1 inflammation, type 2 inflammation, and resolution. Clot and platelet plug formation stop bleeding and establish a source of cytokines that attract primarily innate myeloid cells including neutrophils, eosinophils, and macrophages. The initial immune environment is established by a type 1 response that promotes M1 macrophage polarization, that together with neutrophils perform pathogen killing to re-establish barrier protection and tissue debridement to clear dead tissue for cell recruitment and ECM deposition. Following, Tregs promote immune tolerance and resolution by mediating a switch to a type 2 inflammatory response. M2 polarized macrophages and Tregs facilitate ECM deposition, and secretion of growth factors. Stem cell (SC) and progenitor cell (PC) stimulation via secreted growth factors results in proliferation and recruitment to the re-modeled wound niche, where they facilitate long term tissue remodeling and repair.

Wound healing occurs in four distinct sequential phases of hemostasis, inflammation, proliferation/recruitment, and resolution (Figure 2). Leukocytes of the innate (platelets, neutrophils, eosinophils, basophils, and macrophages) and adaptive (T cells, B cells, natural killer (NK) cells, innate lymphoid cells) immune system are recruited with distinct roles in response to injury. The role of inflammation in reparative healing is first to debride cellular debris and create a provisional matrix to facilitate cellular recruitment (phase I and II). Following, recruited innate and adaptive immune cells then secrete cytokines that stimulate cellular proliferation and tissue remodeling (phase III and IV). These inflammatory responses are guided by distinct type I (pro-inflammatory) and type II (anti-inflammatory) immune regimens.

The inflammatory response to wounding is highly orchestrated to follow four main programs after injury including hemostasis, type 1 inflammation, type 2 inflammation, and resolution. Clot and platelet plug formation stop bleeding and establish a source of cytokines that attract primarily innate myeloid cells including neutrophils, eosinophils, and macrophages. The initial immune environment is established by a type 1 response that promotes M1 macrophage polarization, that together with neutrophils perform pathogen killing to re-establish barrier protection and tissue debridement to clear dead tissue for cell recruitment and ECM deposition. Following, Tregs promote immune tolerance and resolution by mediating a switch to a type 2 inflammatory response. M2 polarized macrophages and Tregs facilitate ECM deposition, and secretion of growth factors. Stem cell (SC) and progenitor cell (PC) stimulation via secreted growth factors results in proliferation and recruitment to the re-modeled wound niche, where they facilitate long term tissue remodeling and repair.

In phase I, hemostasis is achieved within minutes to hours by formation of a platelet plug (Golebiewska and Poole, 2015). Upon activation, platelets release an assortment of cytokines stored in α-granules and dense granules that are involved in the recruitment of circulating inflammatory cells (Zarbock et al., 2007; Golebiewska and Poole, 2015). Following platelet-plug formation, neutrophils and then macrophages are recruited within the first 3–5 days to debride dead cellular debris and for the clearance of potential pathogens that may have invaded during injury (Martin and Leibovich, 2005; Eming et al., 2017). While antigens from cellular debris initially sustain a pro-inflammatory environment that drives debris clearance and host defense, early recruitment of eosinophils and release of type II alarmins act as sources of type II cytokines (e.g., IL4, IL33) that initiate a type II immune profile after completion of phase I and II (Heredia et al., 2013; Molofsky et al., 2015). The release of type II cytokines promotes polarization of innate and adaptive immune cells toward anti-inflammatory phenotypes that induce immune tolerance and release growth factors (such as amphiregulin and TGFβ) that stimulate resident stem and progenitor cell proliferation and recruitment (Burzyn et al., 2013; Eming et al., 2017; Ito et al., 2019; Minutti et al., 2019). Inadequate reparative responses of stem and progenitor cells or immune dysregulation can alternatively recruit myofibroblasts that deposit dense collagen resulting in scar tissue formation or fibrosis (Gudelli and Guo, 2020).

Adaptive immune cells home to the wound site around 5–7 days post injury and orchestrate broad functions. Among adaptive immune cells, Th1/Th17 CD4^+^ T cells, CD8^+^ T-cells, or NK cells may act to sustain a pro-inflammatory (type I) immune environment, while Th2 T cells or Tregs may promote an anti-inflammatory (type II) response. For example, activated Tregs promote M2 macrophage polarization and release growth factors that stimulate reparative healing in the lung, muscle, and brain (Burzyn et al., 2013; Ito et al., 2019; Liu et al., 2019). More recently, innate lymphoid cells (ILC) have been identified as a unique population of lymphocytes that do not express antigen receptors but respond to tissue injury or infection by releasing an array of cytokines to stimulate or resolve inflammation. Among ILCs, ILC1s, ILC2s, and ILC3s, represent counterparts to Th1, Th2, and Th17 T cells (Eberl et al., 2015).

Since the local immune environment is dictated by the stoichiometry of various cytokines that directly shift the balance of inflammation and its resolution, we will next focus on key cytokines associated with each response, with an emphasis on their established roles in tendon repair.

## Cytokines

While wound healing is mediated directly through immune cells, it can be useful to understand the immune programs involved in wound healing through the lens of cytokines, which are more easily quantified and govern the activation and phenotype of recruited immune cells. Here, we discuss selected cytokines that are most heavily studied in the context of wound healing. A more inclusive list of cytokines has been comprehensively reviewed elsewhere (Werner and Grose, 2003; Turner et al., 2014).

### Cytokines Associated With Type I Inflammatory Responses

#### TNFα

Tumor necrosis factor α (TNFα) is initially sequestered to the cell membrane as a transmembrane protein that is then shed as soluble TNFα via ectodomain cleavage by TNFα-converting enzyme (TACE) (Black et al., 1997; Moss et al., 1997). Soluble and membrane-bound TNFα exerts downstream signaling through binding and activation of either TNF receptor 1 (TNFR1) or TNFR2 (Locksley et al., 2001). TNFR1 is expressed broadly with activation of death-domains (TRADD) upon binding of TNFα leading to inflammation (via NFκB, MAPK), apoptosis (via caspase 9), or necroptosis (via RIPK3) (Hsu et al., 1995; Kalliolias and Ivashkiv, 2016). In contrast, TNFR2 is more selectively expressed in certain populations of leukocytes, endothelial, cardiac, and neural cells (Ware et al., 1991; Irwin et al., 1999; McCoy and Tansey, 2008; Kalliolias and Ivashkiv, 2016). TNFR2 is specifically activated by membrane-bound TNFα and signals through TRADD-independent, TNF-receptor associated factor 2 (TRAF2) (Grell et al., 1995). Upon activation, TNFR2 promotes cell survival through MAPK, NFκB, and AKT pathways (Kalliolias and Ivashkiv, 2016; Yang et al., 2018). Therefore, one model of TNF-mediated inflammation hypothesizes that TNFR1 signaling promotes inflammation while TNFR2 maintains homeostasis, with potential implications for tissue regeneration (Yang et al., 2018).

In human Achilles tendon, TNFR1 and TNFR2 was observed by immunohistochemistry in both control and tendinosis samples (Gaida et al., 2012). While some studies show TNFα in samples of human tendinopathy, others do not (Gaida et al., 2012; Ackermann et al., 2013). In animal models of acute tendon injury, TNFα expression increased within the first 9 days of injury and declined within 2 weeks (Morita et al., 2017). *In vitro*, stimulation of tenocytes with TNFα results in increased expression of adhesion proteins and inflammatory cytokines (IL6, IL8), that may add to the inflammatory milieu in tendinopathy, independent of immune cells (Stolk et al., 2017). However, TNFα depletion with etanercept following injury in rats failed to improve or worsen tendon healing measured by peak force (Sandberg et al., 2012). In a rat model of rotator cuff repair, TNFα blockade with infliximab improved tendon to bone repair, suggesting TNFα therapies may differ in efficacy. Indeed, infliximab binds both soluble and membrane bound TNFα more stably, while etanercept has less affinity for TNFα. Furthermore, since infliximab is a complement fixing antibody, cells expressing TNFα undergo lysis, which does not occur with etanercept treatment (Mpofu et al., 2004). Therefore, treatment strategies for modifying TNFα signaling will need to consider differences in pharmaceutical mechanisms of action. Lastly, age-dependent differences in TNFα signaling are also likely to modify treatment. A recent study in a repetitive-use tendinopathy model of rat flexor tendons, observed increased TNFα in younger rats (Kietrys et al., 2012).

#### IL1β

An acute phase reactant, interleukin-1β (IL1β) is a potent inflammogen that is critical for response to injury, infection, and malignancy (Lopez-Castejon and Brough, 2011; Bent et al., 2018). Along with IL1α, IL18, IL33, IL36α/β/γ, IL1β is a member of the IL1 cytokine family (Garlanda et al., 2013). Unlike the preformed alarmins IL1α and IL33, precursor-IL1β (pre-IL1β) is secreted as a 31 kDa proprotein in response to toll-like receptor (TLR) activation, complement signaling, or secondary cytokine (TNFα, IL1α) stimulation (Lopez-Castejon and Brough, 2011; Garlanda et al., 2013). Following cleavage by caspase-1, pre-IL1β is secreted extracellularly in its active form (Thornberry et al., 1992). Among the IL1 cytokine family, there are 10 distinct receptors (IL1R1 thru IL1R10) (Boraschi and Tagliabue, 2013). Following binding of IL1β to IL1R1, the ligand-receptor dimerizes with IL1R3, bringing together Toll-interleukin receptor (TIR) domains and binding of MyD88 with downstream signaling through activation of MAPK or NFκB (Dinarello, 2018).

In clinical samples of tendinopathy, the presence of IL1β is ambiguous. Microdialysis of human Achilles tendon 2 weeks following repair of acute rupture showed no significant difference compared to uninjured tendons (Ackermann et al., 2013); however, early stage subscapularis tendinopathy showed increased expression of IL1β (Abraham et al., 2019). In preclinical studies, IL1β is generally observed in the early stages of tendon injury with stimulation leading to increased expression of IL6 and IL8 (Morita et al., 2017; Stolk et al., 2017). In rats, *in vivo* fatigue testing of patellar tendons resulted in increased expression of MMP13 and IL1β, with observed microstructural damage (Sun et al., 2008). Interestingly in bioartificial tendon constructs, IL1β decreased ultimate tensile strength and elasticity (Qi et al., 2006a). In tenocytes, silencing of IL1β signaling decreased load-dependent MMP13 expression, suggesting that IL1β may drive MMP13-mediated ECM degradation (Corps et al., 2004; Qi et al., 2006a, b; Sun et al., 2008). In mouse tenocytes, constitutively active NFκB signaling sensitized cells to IL1β, with increased expression of TNFα, prostaglandin-endoperoxide synthase 2 (PTGS2), granulocyte colony-stimulating factor (G-CSF) and CXCL2 following IL1β stimulation (Abraham et al., 2019). Knockdown of NFκB signaling desensitized tenocytes to IL1β insult (Abraham et al., 2019). More directly, deletion of NFκB in mice resulted in increased apoptosis of myofibroblasts indicating NFκB signaling promotes myofibroblast survival (Best et al., 2020). In humans, tenocytes isolated from tendon biopsies 2 to 4 years post-treatment demonstrated increased expression of IL6, IL8, and activated fibroblast marker podoplanin (PDPN) when stimulated with IL1β, in comparison to IL1β-treated healthy controls (Dakin et al., 2017). Together, these data indicate that tendon healing may be impeded in the background of persistent NFκB signaling via increased IL1β sensitivity, and that inflammation is a feature of chronic tendinopathy.

#### IFNγ

Interferon gamma (IFNγ) is a cytokine involved in orchestrating the innate and adaptive immune response to bacterial, fungal, and viral pathogens (Bhat et al., 2018). IL12 and IL2 costimulation of Th0 (naive) T cells drives differentiation into Th1 cells, marked by secretion of IFNγ (Del Prete et al., 1994; Spellberg and Edwards, 2001). In addition, IFNγ can be produced by ILC1s and CD8^+^ cytotoxic T cells (CTLs) (Klose et al., 2014; Bhat et al., 2017). IFNγ binds to the IFNγ Receptor Complex (IFNGR), which leads to receptor dimerization and downstream expression of interferon stimulated genes (ISGs) and interferon response factors (IRF) via JAK-STAT signaling (Bhat et al., 2018). IRFs and ISGs act broadly to promote innate and adaptive type I inflammatory responses, including increasing inflammatory cytokine production, nitric oxide synthase (important to generate free radicals to kill pathogens), antigen presentation, NK cell activity, and leukocyte migration (Volk et al., 1986; Martin et al., 1994; Bhat et al., 2018). Moreover, recent data indicates that IFNγ signaling leads to broad transcriptional and epigenetic signatures that favors differentiation of immune cells into a type I phenotype (e.g., M1-like macrophages and Th1 T cells) (Ivashkiv, 2018). Importantly, secretion of IFNγ is also important for negative regulation of type II inflammation (Gajewski and Fitch, 1988; Gajewski et al., 1988). For example, binding of IFNγ leads to phosphorylation of STAT1 that inhibits IL4 mediated STAT6 signaling via SOCS1 (Zhao et al., 2019). Conversely, phosphorylation of STAT6 via IL4, inhibits downstream STAT1 signaling via inhibition of IRF1, thereby blocking upstream IFNγ signaling (D’andrea et al., 1993; Ohmori and Hamilton, 1997; Ito et al., 1999; Zhao et al., 2019).

The role of IFNγ in tendinopathy and healing remains poorly characterized. While some studies show little to no observable levels of IFNγ in acute Achilles tendon rupture 2 weeks postoperatively, others show increased expression of IFNγ in early stage tendinopathy samples of human subscapularis (Ackermann et al., 2013; Abraham et al., 2019). These inconsistencies may be due to differences in mechanism of injury, tendon origin (Achilles vs. rotator cuff), or expression level (protein vs. gene expression). Moreover, a study comparing gene expression of cytokines across the proximal, middle, and distal torn edge of the supraspinatus tendon found significant differences in IL1β, IFNγ, IL4, and IL13 with corresponding changes in collagen I expression (Fabiś et al., 2014). Interestingly, IFNγ (type I cytokine) expression was greatest at the distal edge, and lowest at the proximal edge, while IL4 (type II cytokine) had an inverse expression profile. This suggests that local immune profiles may play mutually exclusive and distinct roles in different zones of the injured tendon that might be lost in aggregate profiling. The role of IFNγ in tendon healing remains unclear. *In vitro*, tenocytes isolated from tendinopathic and ruptured Achilles tendon have increased expression of interferon response cytokines when stimulated with IFNγ, compared to tenocytes derived from healthy control tendons (Dakin et al., 2018). However, given that the expression of cytokines and inflammatory genes were more dramatic when stimulated with other inflammatory cytokines (IL6, IL1β), it is possible that IFNγ may play a more dominant role in recruited immune cells vs. directly in tenocytes (Stolk et al., 2017; Dakin et al., 2018).

#### IL6

IL6 is a cytokine that typically signals infection or tissue injury, and stimulates production of acute phase reactants (e.g., fibrinogen, C-reactive protein, serum amyloid A, hepcidin) to promote host defenses (Castell et al., 1989). Paradoxically, the beneficial role of IL6 in tissue protection and healing has also been observed. In rats, IL6 administration prior to CCL4 challenge with partial hepatectomy promoted liver regeneration and survival (Tiberio et al., 2007, 2008). Similarly in the gut, IL6 mediated STAT3 signaling is required for epithelial healing in a mouse model of colitis (Grivennikov et al., 2009). Binding of IL6 to its receptor (IL6R), facilitates recruitment and dimerization of glycoprotein-130 (gp130) which leads to downstream JAK-SHP2-MAPK, JAK-AKT, and JAK-STAT3 signaling (Tanaka et al., 2014). IL6 has no appreciable affinity for gp130, therefore signaling requires expression of IL6R which is not ubiquitously expressed in all cells (Jostock et al., 2001; Wolf et al., 2014). Interestingly, recent studies have demonstrated a non-canonical mechanism of IL6 signaling known as trans-signaling. In trans-signaling, IL6R is shedded in a soluble form (sIL6R) following cleavage by ADAM17. This soluble form can then bind IL6 to stimulate gp130 signaling in cells that do not natively express IL6R (Rose-John, 2012). It is hypothesized that the paradoxical differences in inflammatory vs. reparative mechanisms of IL6 signaling may be explained by trans- vs. canonical IL6 signaling (Rose-John, 2012; Galun and Rose-John, 2013). In a series of elegant experiments, the contribution of trans- and canonical IL6 signaling was delineated by expressing the extracellular soluble domain of gp130 (sgp130), which was used to selectively block IL6 trans-signaling by binding to and sequestering sIL6R (Jostock et al., 2001; Rose-John, 2012). Using a cecal ligation and puncture (CLP) model, it was shown that blockade of IL6 trans-signaling but not global IL6 led to improved survival following CLP, indicating that trans-signaling may largely contribute to the inflammatory arm of IL6 signaling while canonical-membrane bound IL6 signaling may be largely responsible for improved healing (Barkhausen et al., 2011; Rose-John, 2012).

In T cells, the role of IL6 is similarly complex. IL6 promotes differentiation of naive Th0 cells into Th17 cells and inhibits Treg differentiation. IL6 also inhibits IFNγ-mediated differentiation of Th1 cells by inducing expression of SOCS1. In contrast, IL6 induces IL4 expression in an autocrine fashion that stimulates Th2 differentiation. Taken together, the role of IL6 in wound healing can be both reparative and inflammatory and mitigating factors will likely continue to be elucidated.

Given the wide role of IL6, there is much interest in its role in tendinopathy and tendon healing. In human specimens of acute Achilles tendon or rotator cuff rupture, IL6 expression by protein microdialysis and RNA gene expression was significantly increased when compared to uninjured tendons (Nakama et al., 2006; Ackermann et al., 2013). Importantly, inflammatory cytokines TNFα and IL1β are known inducers of IL6 expression in human tenocytes (Tsuzaki et al., 2003; John et al., 2010). However, in specimens of chronic tendinopathy, there is little evidence of elevated IL6, suggesting a more prominent role of IL6 in healing as a response to inflammation vs. as a driver (Morita et al., 2017). In IL6^–/–^ mice, tendons developed normally, however tendon injury results in impaired mechanical restoration and collagen organization (Lin T. W. et al., 2005; Lin et al., 2006). More broadly, it was proposed that IL6 may play a role in tendon homeostasis in addition to its role in response to acute tendon rupture. Following exercise, peritendinous IL6 concentration was increased 100-fold relative to serum concentration in human Achilles tendon (Langberg et al., 2002; Andersen et al., 2011; Gump et al., 2013). Consistent with this, cyclic loading of bovine tendon fascicles explants or human tenocytes *in vitro* led to an increase in IL6 expression (Skutek et al., 2001; Legerlotz et al., 2013). Interestingly, Achilles tendon production of procollagen propeptide increased in response to peritendinous injection of IL6 in humans (Andersen et al., 2011). Expression of IL6 was also increased in mouse Achilles tendons treated with collagenase (Ueda et al., 2019). In sum, IL6 plays an important role in tendon healing; however, the mechanisms by which IL6 signals tendon homeostasis and repair remain unclear.

### Cytokines Associated With Type II Anti-inflammatory Responses

#### IL4

Upon stimulation, type 2 innate and adaptive cells (e.g., eosinophils, Th2 T cells, ILC2s) release a milieu of type 2 cytokines, most notably IL4, IL5, and IL13. Among these, IL4 is a potent inducer of type 2 polarization and can suppress IFNγ-producing CD4^+^ T cells that promote type 1 polarization (Tanaka et al., 1993). Classically, induction of type 2 inflammation following parasitic infiltration results in IL4 production which promotes Th2 activation, IgE class switching, and eosinophil activation that cooperatively function in parasite elimination (Spellberg and Edwards, 2001; Henry et al., 2017). In parallel, IL4 functions broadly to activate resident macrophages and fibroblasts to repair damaged tissue resulting from parasitic insult. Since parasitic infiltration most commonly occurs via mucosal entry, much of what is known regarding IL4 mediated wound healing comes from studies of the mucosal lungs and gut. In the lungs, IL4 signaling following helminth infection results in phenotypic polarization of macrophages to an alternatively activated (M2) fate (Chen et al., 2012). M2 macrophages facilitate tissue repair with production of growth factors (TGFβ1, IGF1, VEGF, PDGF, and RELM), and suppress type 1 inflammatory cytokines (e.g., IL17A) via IL10 (Chen et al., 2012; Wynn and Vannella, 2016). More recently, it was revealed in the lung that surfactant protein A enhanced IL4 sensing in macrophages suggesting tissue specific regulatory loops that promote IL4 mediated tissue repair (Minutti et al., 2017). In the gut, conditioned media from IL4-stimulated M2 macrophages improved epithelial barrier integrity in a mouse model of inflammatory colitis (Jayme et al., 2020). Consistent with these organ systems, the major contributor of IL4 mediated tissue repair is dependent on macrophage polarization to an M2 fate, with subsequent scavenging of apoptotic bodies and growth factor release (Bosurgi et al., 2017). In non-lymphoid muscle, however, production of IL4 from infiltrating eosinophils directly controls fate switching of resident fibroadipogenic progenitor cells to promote myogenesis and inhibit adipogenesis (Heredia et al., 2013).

Since IL4 is critical for wound healing in various tissues, recent studies have sought to elucidate potential roles for IL4 in tendon repair. In human samples of torn supraspinatus tendons, IL4 and IFNγ are counter-expressed with high expression of IL4 at the proximal segment of the torn tendon that decreases distally. Interestingly, cell proliferation and collagen I expression follows a pattern of expression similar to IL4 suggesting shared responses to injury (Courneya et al., 2010). In IL4^–/–^ mice, only minor differences in stress relaxation and collagen organization were observed after tendon injury, which may suggest potential compensation by other cytokines such as IL13 and IL10 (Lin et al., 2006). Alternatively, since the pro-regenerative effect of IL4 is likely mediated via macrophages, impaired IL4:M2 macrophage polarization associated with aging may explain minimal differences between WT and IL4^–/–^ mice (Mahbub et al., 2012). In bone fracture healing, aging was associated with increased activation of M1 macrophages associated with increased expression of TNFα following stimulation, compared to macrophages from younger mice. Moreover in adult mice, blockade of macrophage recruitment improved bone fracture healing (Gibon et al., 2016; Clark et al., 2020). These studies, conducted in bone, suggest that IL4:macrophage polarization may be restricted neonatally, with aging resulting in an M1 macrophage bias that is associated with poor functional healing. While M2 macrophages are known to improve tendon healing, future studies to compare differences in neonatal and adult healing may shed further light on immune mechanisms that promote regeneration (Mauro et al., 2016).

#### IL5

IL5 is a type 2 cytokine that plays a critical role in the activation and survival of eosinophils (Adachi and Alam, 1998). Binding of IL5 ligand to the cognate receptor drives eosinophil activation/degranulation and survival. Activation of Lyn, Syk, and JAK2 by IL5 promotes survival, while Raf1 kinase is primarily involved in degranulation and expression of adhesion proteins (van der Bruggen et al., 1995; Yousefi et al., 1996; Pazdrak et al., 1998). Clinically, IL5:eosinophil activation is classically associated with atopic diseases, most notably asthma (Adachi and Alam, 1998). However more recently, several studies have uncovered novel roles for eosinophils in wound healing. Shortly after injury, recruited eosinophils assist in thrombosis and platelet plug formation to assist in hemostasis (Coden and Berdnikovs, 2020). Additionally, eosinophils exposed to fibrogen undergo degranulation, releasing polarizing type 2 cytokines that promote pro-regenerative M2 macrophages (Heredia et al., 2013). In muscle following injury, eosinophil deficient mice exhibit increased fatty degeneration with poor debris clearance, which was shown to be mediated via FAP:IL4 signaling (Yang et al., 1997; Heredia et al., 2013). While eosinophil recruitment is critical for restorative tissue repair, excessive eosinophilia can also be deleterious to healing. In mice, overexpression of IL5 with characteristic eosinophilia showed delayed epithelial wound healing with impaired collagen deposition and gap closure (Yang et al., 1997). Therefore, it is likely that pro-regenerative healing requires a well orchestrated cascade of signals that must be properly balanced to promote healing. Currently, there are no studies that have investigated the role of IL5 in tendon repair. However, given the role of IL5 in wound healing in other systems, it is likely that IL5 is also present in tendon injury. Future experiments that investigate the role of IL5 will provide valuable insights into the immunobiology of tendon healing.

#### IL13

Similar to IL4, the role of IL13 in tissue healing has been heavily studied. In parasitized organs, release of IL13 by Th2 cells promotes activation of tissue resident fibroblasts to polarize macrophages toward a M2 phenotype (Murray and Wynn, 2011; O’Reilly et al., 2016). In contrast, following wounding in the lung, alveolar epithelial cells secrete IL13 suggesting differences in upstream mediators that distinguish septic vs. aseptic inflammation (Allahverdian et al., 2008). IL13 signals via an IL4Rα and IL13Rα1 heterodimer to activate the transcription factors STAT3 and STAT6, which act broadly to activate healing responses in the gut, neonatal heart, and lung (Hershey, 2003; Allahverdian et al., 2008; Yang et al., 2019; Zhu et al., 2019). In the gut, IL13 is produced by ILC2s in response to parasitic infection or tissue damage. Interestingly, stimulation of Lgr5^+^ intestinal crypt stem cells by IL13 results in expression of Foxp1 which stabilizes nuclear translocation of β-catenin, thereby driving intestinal stem cell proliferation and self-renewal (Bouchery et al., 2019; Zhu et al., 2019). Similarly, in neonatal heart regeneration, activation of ERK/AKT signaling by IL13 drives cardiomyocyte proliferation that is blocked in IL13^–/–^ mice (Wodsedalek et al., 2019). In the lung, production of IL13 by Tregs results in macrophage polarization toward a Ly6c^lo^ (reparative) phenotype and secretion of amphiregulin, which drives proliferation of type II alveolar epithelial cells (Liu et al., 2019).

Given the broad role of IL13 in tissue repair, it is unsurprising that several studies also implicate IL13 in tendon repair. Histological assessment and gene expression of IL13 in human tendinopathy samples demonstrated robust presence of IL13 indicating clinical relevance (Fabiś et al., 2014; Akbar, 2018). *In vitro* stimulation of human tenocytes with IL13 demonstrated a twofold increase in proliferation, in a dose-responsive fashion (Courneya et al., 2010; Akbar, 2018). Future studies may further determine whether IL13 mediates tendon healing via macrophage polarization, as has been shown in other tissues.

#### IL33

Part of the IL1 cytokine family, IL33 is a type 2 cytokine that plays an important role in innate and adaptive immunity. Despite the relatively recent discovery of IL33, numerous studies have demonstrated broad and robust roles of IL33 in homeostasis and disease (Liew et al., 2016; Jin et al., 2020). An alarmin, IL33 is produced by tissue resident stromal cells and sequestered in the nucleus. Following damage induced necroptosis, release of IL33 triggers downstream responses in ILC2s, macrophages, Tregs, and other innate immune cells (Liew et al., 2016; Jin et al., 2020). IL33 binds to the suppression of tumorigenicity 2 (ST2) receptor which leads to downstream activation of NFκB and MAPK signaling via activation of MYD88, IRAK1/4, and TRAF6 (Schmitz et al., 2005; Liew et al., 2016). Non-canonically, nuclear IL33 can act in an intracrine fashion by binding to chromatin or inactivating transcription factors to modulate gene expression (Carriere et al., 2007; Ali et al., 2011). Interestingly, this second layer of IL33 signaling has been shown to suppress IL1β mediated expression of target genes IκBα, TNFα, and CREL by sequestration of the p65 subunit involved in NFκB signaling (Ali et al., 2011).

With respect to wound healing, in the past 5 years several studies showed that IL33 is critical for tissue repair. Broadly, release of IL33 following injury results in polarization or recruitment of type 2 immune cells that release growth factors to stimulate resident stem and progenitor cell proliferation. Interestingly, Tregs seem to have an essential role in mediating IL33-driven tissue repair in the gut, brain, muscle, and lung (Burzyn et al., 2013; Monticelli et al., 2015; Ito et al., 2019; Liu et al., 2019). In all four systems, activation via the ST2 receptor results in release of amphiregulin from Tregs that promotes tissue repair, suggesting shared healing programs across tissues with diverse origins. In the gut, IL33 also stimulates the release of amphiregulin by ILC2s which promotes intestinal stem cell proliferation (Monticelli et al., 2015). In the lung and brain, IL33 seems to be dependent on a Treg:macrophage axis. Treg activation via IL33 was critical for functional recovery and promoted polarization of pro-regenerative Ly6c^lo^ macrophages and neuroprotective microglia in the lung and brain, respectively (Ito et al., 2019; Liu et al., 2019). Recently, dysregulated IL33 signaling associated with aging was revealed as a contributor to poor muscle healing in mice. In aged (>20 month) mice, decreased IL33 signaling was associated with impaired Treg recruitment and increased fatty infiltration, resulting in poor muscle repair (Kuswanto et al., 2016). These data highlight differences in immunity with age that may contribute to regenerative vs. fibrotic healing. In tendon, the role of IL33 in repair has not been fully characterized. However, IL33 is present in human samples of tendinopathy (Millar et al., 2015). Strikingly, in contrast to other tissues, knockout of IL33 or ST2 resulted in improved tendon healing in adult mice. Mechanistically, IL33 in adult mice promoted a switch from type I to type III collagen, resulting in mechanically inferior tendon, which was rescued with deletion of IL33 signaling (Millar et al., 2015). In chronic parasitic infections that invoke a sustained type 2 response, chronically elevated IL33 contributes to liver fibrosis (Peng et al., 2016). Therefore, it is possible that while IL33 may be required for tissue repair, if it is not resolved, sustained IL33 signaling may paradoxically contribute to fibrosis. Future studies that further elucidate the functions of IL33 signaling in tendon healing will undoubtedly add to the understanding of tissue regeneration and disease.

#### TGFβ

In mammals, transforming growth factor beta (TGFβ) exists in three isoforms (TGFβ1, TGFβ2, TGFβ3) which together have diverse roles in proliferation, differentiation, apoptosis, and migration that are covered with great detail in other excellent reviews (Massagué, 1998; Derynck and Budi, 2019). TGFβ is produced by cells in an inactive state where it is bound to latency associated peptide (LAP) forming a latency complex (Worthington et al., 2011). Large latency complexes (LLC) bind to ECM, where they can be readily cleaved and available to receptive cells. In this manner, abundant sources of TGFβ are readily available with minimal transcriptional or translational latency. Following injury, ECM disruption liberates TGFβ ligands which can then stimulate downstream signaling. Importantly, activated TGFβ has important roles in immune modulation that may have implications in wound healing.

The role of TGFβ in inflammation is generally considered to promote immune tolerance or decrease inflammation (Ulloa et al., 1999; Park et al., 2007). In conjunction with naive CD4^+^ T cell stimulation, TGFβ drives expression of Foxp3 that results in induction of Treg fate (Lu et al., 2010). Tregs provide anti-inflammatory signals (e.g., IL10) that prevent overactive inflammation (Palomares et al., 2014). Moreover, there is extensive counter-regulation among TGFβ and IFNγ that regulate the balance between type 2 (e.g., Th2, M2 macrophage) vs. type 1 polarity (e.g., Th1, M1 macrophage) of leukocytes. Repression of Tbet by TGFβ via MEK/ERK signaling suppresses IFNγ, while upregulation of SMAD7 inhibits SMAD3/6 activation by TGFβ (Ulloa et al., 1999; Lin J. T. et al., 2005; Park et al., 2007). In tendon, latent TGFβ is present in the tendon matrix and is critical for tendon maintenance and regeneration in neonatal mice (Maeda et al., 2011; Kaji et al., 2020; Tan et al., 2020). However, while it is clear that TGFβ signaling in Scx^+^ tenocytes is critical for tendon healing as well as scar and adhesion formation (Katzel et al., 2011; Goodier et al., 2016; Kallenbach et al., 2021), it is unclear whether TGFβ released following ECM disruption during injury plays an additional role in immune modulation.

## Clinical Evidence for Tendon Immunomodulation Therapies

Although tendon injuries are one of the most common musculoskeletal injuries, the guidance regarding clinical management is immature. In the treatment of acute tendon ruptures, re-rupture rate was significantly decreased among operative vs. non-operative treatment; however, operative treatment was associated with greater complications (Deng et al., 2017; Ochen et al., 2019). The use of eccentric physical therapy, shockwave ultrasound, and nitroglycerin among other therapies in the management of tendon repair remains under active investigation and is reviewed elsewhere (Gambito et al., 2010; Sussmilch-Leitch et al., 2012; van der Vlist et al., 2021). One intriguing avenue for immunomodulation may be via delivery of cells with anti-inflammatory properties such as mesenchymal stem cells (MSCs). Recently, it was proposed that MSC-secreted exosomes may contain factors that regulate the immune response during tendon repair (Chamberlain et al., 2019). As this is still an emerging area of research, we focus on pharmacologic interventions that have been tested clinically in this review. Among pharmacologic immune modulators, non-steroidal anti-inflammatory (NSAID) and corticosteroid injections are commonly used in the treatment of tendon injury (Hart, 2011; van den Bekerom et al., 2014). However, the biologic rationale for their use is still debated. It is now recognized that various immune programs are elicited in tendinopathy, and broad suppression using NSAIDs or corticosteroids may either be beneficial or deleterious depending on timing, pathology, and immune environment. Therefore, more selective immune modulatory drugs may provide additional insight into the mechanisms of reparative tendon inflammation.

### NSAIDs

NSAIDs inhibit prostaglandin synthesis by inactivation of cyclooxygenase-I (COX1) or COX2 enzymes. Prostaglandins produced by COX1 are involved in platelet aggregation, gastric mucosal integrity, and renal blood flow, while COX2 derived prostaglandins contribute to pain and inflammation (FitzGerald, 2003; Ghosh et al., 2019). Therefore, the result of non-specific COX inhibition is decreased inflammation and analgesia but with non-desirable secondary effects. Therefore, selective COX2 inhibitors (e.g., celecoxib) have been developed in an effort to selectively decrease pain and inflammation. *In vitro*, treatment of tenocytes with NSAIDs has been reported to suppress proliferation and migration, but was also associated with increased collagen I expression (Su and O’Connor, 2013). In rat models of Achilles tendon tear, post-operative treatment for 14 or 18 days with both indomethacin (COX1 and COX2 inhibition) and celecoxib (COX2 inhibition) improved tendon tensile strength (Forslund et al., 2003). However, in a systematic review of randomized control trials in humans, selective COX2 inhibition was consistently associated with impaired tendon, labrum, and ligamentous healing, suggesting a requirement for inflammation in reparative healing (Ghosh et al., 2019). While the role of COX2 related prostaglandins in tendon repair is unclear, timing appears to be a critical factor. In rats, treatment with parecoxib (COX2 inhibitor) for the first 5 days following tendon transection impaired tendon repair, while delayed treatment with parecoxib 6–14 days after transection improved tendon repair (Virchenko et al., 2004). Non-selective NSAIDs show conflicting results probably due to differences in COX1 vs. COX2 inhibition and treatment timing. Given the complex nature of prostaglandins to tissue repair, further understanding of the cellular and molecular mechanisms of prostaglandin mediated tissue repair are required to better understand how to fine-tune pharmaceutical interventions.

### Steroids

Corticosteroids are a class of hormones that are endogenously produced by the adrenal cortex and are critical in homeostasis for a wide range of processes. Synthetic corticosteroids (e.g., dexamethasone) are commonly used for their anti-inflammatory effects. Mechanistically, corticosteroids are lipophilic molecules that diffuse across the plasma membranes and bind to intracellular glucocorticoid receptors (GR) (Sivapriya Ramamoorthy, 2016). Following binding, corticosteroid-GR complexes migrate intra-nuclearly and can directly modulate gene expression via glucocorticoid response elements (GREs) (Escoter-Torres et al., 2019). In a systematic review of pre-clinical models, corticosteroids administration was associated with inferior mechanical properties of tendons (Dean et al., 2014). *In vitro*, steroids decreased cell viability and collagen synthesis (Dean et al., 2014). However, timing of steroid injections might be critical to therapeutic effect. Similar to NSAID administration, dexamethasone treatment in rats day 0–4 following Achilles tendon transection resulted in impaired healing while treatment on days 5–9 following transection improved tendon repair (Blomgran et al., 2017). In humans, randomized control trials of corticosteroid injection for treatment of tendinopathy were consistently associated with improved pain within the first 12 weeks following injection but was associated with impaired or no improvement in the intermediate (13–26 weeks) to long term (≥52 weeks) periods after injection (Efficacy and safety of corticosteroid injections and other injections for management of tendinopathy: a systematic review of randomized controlled trials, Coombes et al., 2010). Strikingly, preoperative administration of corticosteroids prior to rotator cuff repair surgery was associated with increased risk of postoperative infection and revision that increased with more frequent dosing or shorter time interval from administration to surgery (Puzzitiello et al., 2020).

Taken together, while the clinical utility of corticosteroids and NSAIDs are limited due to broad off-target effects, the literature demonstrates a strong role for inflammation in tendon repair. The development of more targeted immunobiologics may promote reparative inflammation while avoiding the harmful effects of steroids and NSAIDs.

### Targeted Immune Modulators

Since the development of the first disease modifying antirheumatic drugs (DMARDs) in the 1950’s (hydroxychloroquine and azathioprine), selective immune modulating drugs continue to offer novel treatments for various inflammatory pathologies. With respect to tendon repair, the benefit of DMARD drugs is highly variable with some appearing to impair tendon repair or in some circumstances increase likelihood of rupture. However, since most patients treated with DMARDs have underlying rheumatologic disease, it is often challenging to decouple the positive or negative effects related to the drug treatment, vs. primary disease. To date, there are no high quality randomized controlled trials that investigate the role of DMARDs in tendon repair among patients without rheumatologic disease.

### Platelet-Rich Plasma

Platelet-rich plasma (PRP) is an autologous derivative of whole blood plasma that is enriched for platelets, growth factors, and fibrin matrix (Alves and Grimalt, 2018). PRP was first denoted in the 1970’s by hematologists for the treatment of thrombocytopenia and was then later leveraged in dentistry and maxillofacial surgery for its hemostatic properties (Whitman et al., 1997). More recently in the field of orthopedics, there has been vigorous interest in the use of PRP for various treatments (Hsu et al., 2013). However, there is no consensus regarding PRP treatment with minimal reliable evidence to guide clinical use. For the treatment of tendinopathy, the evidence is mixed. Systematic reviews and meta-analyses demonstrate decreased long-term pain in patients with rotator cuff injuries or lateral epicondylitis when treated with PRP, but also show evidence of bias with a large degree of heterogeneity among studies (Chahla et al., 2017). A more recent high-quality randomized control trial of a single treatment of PRP among adults with acute Achilles tendon rupture showed no difference in primary or secondary outcomes when compared to placebo (D’Hooghe and Vinagre, 2020). Unsurprisingly, heterogeneity in PRP related to donor, preparation, and activation are all likely contributors to differences in therapeutic efficacy. Indeed, a comparison of PRP from various commercial collection methods showed dramatic differences in PRP composition, with notable differences in cellular and growth factor concentrations (Castillo et al., 2011; Magalon et al., 2014). Varying preparation can enrich leukocytes yielding leukocyte-rich (LR-PRP) or leukocyte-poor (LP-PRP) PRP, which may have different therapeutic effects. Pre-clinical data in rabbits found that treatment with LR-PRP improved tendon quality on histology with increased inflammation in uninjured healthy patellar tendons (Dragoo et al., 2012).

In a double-blind randomized control trial, athletes with ≥6 months patellar tendinopathy were treated with a single dose of LR-PRP, LP-PRP, or saline in combination with an exercise rehabilitation program. In contrast to another trial that demonstrated moderate benefit of LR-PRP, no significant difference was observed among the three groups (Dragoo et al., 2014; Scott et al., 2019). Since PRP is an autologous source, baseline differences in study participants and differences in control arms comparisons (dry needle stick vs. saline injection), are likely sources of bias that may account for differences in outcomes.

Additional formulations of PRP continue to be developed with various types of activation and collection (Landesberg et al., 2005; McCarrel et al., 2014; Zhang et al., 2019). Currently, there is no consensus regarding the characterization of PRP, since the immune landscape during tissue repair is immature and remains poorly characterized. Future studies that further define the role of inflammation in tissue repair can aid in engineering of PRP formulations that produce pro-regenerative inflammatory components.

## Discussion

While there has been significant research in understanding tendon healing, the role of inflammation remains poorly understood. Unlike other tissues (such as lungs, gut, and muscle) tendons heal by scarring with poor functional outcomes. Among regenerative tissues, inflammation is critical for normal tissue repair. Therefore, modification of the immune landscape during tendon injury presents an opportunity to promote a pro-regenerative immune milieu. One challenge in better identifying pro-regenerative inflammatory responses in tendon healing, is that tendon healing is non-regenerative in nature, with scar tissue formation and poor restoration of functional outcomes. Therefore, rescuing tendon regeneration by either inhibiting or upregulating individual cells or cytokines, is a more challenging task than loss of function experiments. Despite this, immune modulation presents a safe and tractable strategy to improve tendon healing with many FDA-approved immune modulatory drugs and therapies currently available.

**FIGURE 3:**
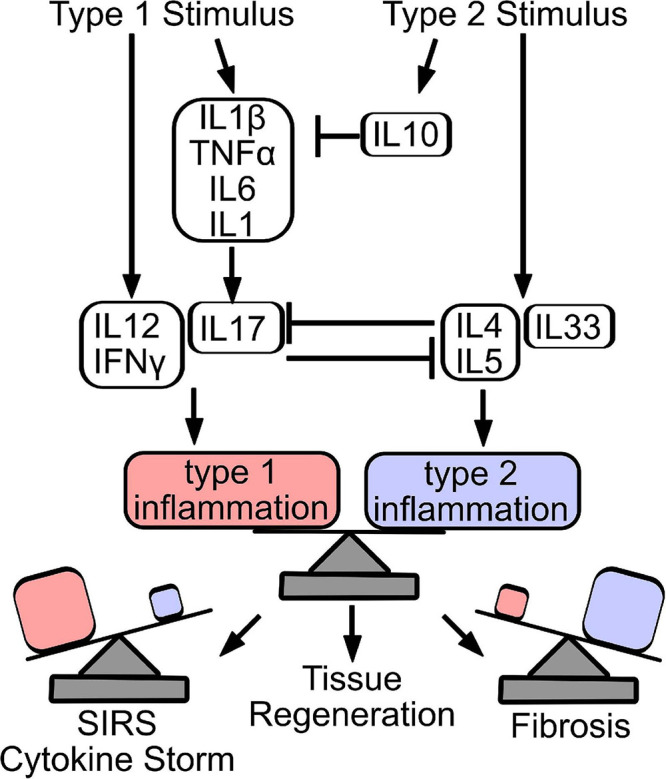
Counter-regulation and balance of type 1 and type 2 inflammatory responses dictate repair outcome. Stimulation of a type 1 immune response produces cytokines that stimulate innate (IL1β, TNFα, IL6, and IL1) and adaptive (IL12, IFNγ, and IL17) immune response. Similarly, release of IL4, IL5, and IL33 induces a type 2 immune response, while IL10 acts primarily to resolve acutely produced type 1 cytokines. Type 1 and type 2 cytokines counter-regulate, to establish the predominant immune signature. Importantly, effective tissue regeneration depends on the balance of inflammatory programs, where overactive type 1 inflammation can result in sudden inflammatory response syndrome (SIRS) or cytokine storm, while excessive type 2 inflammation drives tissue fibrosis.

To realize this goal there are several open questions that face the orthopedic community. First, it is unclear mechanistically how immune cells or cytokines mediate tendon repair *in vivo*. Does the immune environment primarily function to prepare a supportive niche, or do immune cells secrete/activate growth factors that directly stimulate stem and progenitor cell proliferation and recruitment? Second, it is clear that timing plays a critical role. Early or delayed administration of immune modulatory drugs following injury drastically affects tendon repair outcomes. Imaging or biochemical assays that can stage the timing of inflammation following injury are essential to determine when to provide appropriate interventions. Induction of a type II response in lieu of an early type I response impairs debridement and tissue clearance, while prolonged type I or type II response can lead to cytokine storm or fibrosis, respectively. Therefore, it is important to understand the interplay and timing of type I and type II inflammatory programs to adequately balance responses to promote reparative inflammation (Figure 3).

Stimulation of a type 1 immune response produces cytokines that stimulate innate (IL1β, TNFα, IL6, and IL1) and adaptive (IL12, IFNγ, IL17) immune response. Similarly, release of IL4, IL5, and IL33 induces a type 2 immune response, while IL10 acts primarily to resolve acutely produced type 1 cytokines. Type 1 and type 2 cytokines counter-regulate, to establish the predominant immune signature. Importantly, effective tissue regeneration depends on the balance of inflammatory programs, where overactive type 1 inflammation can result in sudden inflammatory response syndrome (SIRS) or cytokine storm, while excessive type 2 inflammation drives tissue fibrosis.

Importantly, while we have discussed type I and II inflammatory responses as dichotomous archetypes to explain wound healing responses, in reality, *in vivo* immune phenotypes and responses lie along a continuum. Heterogeneous contributions of type I and type II inflammation can reflect responses along the continuum, or can reflect an intermediary transition state from one program to the other. Unlike static states of repair or destruction, immune responses to wound injury are more likely to represent dynamic processes that are primed by constantly changing immunologic stimuli, cytokines, and stresses (Mujal and Krummel, 2019). Moreover at the cellular level, next generation sequencing studies in tendon have demonstrated a continuum of cellular phenotypes that contribute to wound healing (Ackerman et al., 2021; Akbar et al., 2021).

Lastly, while much of the work teasing the role of inflammation in tendon repair has been conducted in healthy mice, patients may have differences in underlying inflammation. For example, it is known that metabolic disease (i.e., type 2 diabetes mellitus) can predispose patients to a baseline inflammatory environment (Wellen and Hotamisligil, 2005). Therefore, it is also important to interrogate reparative inflammation through a diverse lens to tease background contributions related to pathology, but also related to sex and age. Since we have previously shown tendon regeneration after injury in the neonatal mouse (Howell et al., 2017), comparing age-dependent differences in immune response between neonates and adults may aid in identification of pro-regenerative immune programs in tendon. Progress in this endeavor will inform the translation of immune modulatory drugs that can improve tendon repair.

## Author Contributions

VA and AH contributed to conception, writing, and editing of this review. Both authors contributed to the article and approved the submitted version.

## Conflict of Interest

The authors declare that the research was conducted in the absence of any commercial or financial relationships that could be construed as a potential conflict of interest.
